# Fog and Smoke

**Published:** 1905-01-07

**Authors:** 


### Fog and Smoke.

The pall of smoke-laden fog which covered the metropolis during many days of the past Christmas season, and which, although somewhat less smoke-laden, extended itself to many parts of the kingdom, constituted an evil, which was of much greater magnitude than many others which we take more pains to bring under control. The cost in human life of a dense urban fog is always considerable, partly from the accidents which it occasions and partly from its effects upon the respiratory organs ; and it is probable that no correct estimate of this cost can be formed except from data extending over several weeks after the occurrence. The money cost incidental to the deposit and the subsequent removal of dirt is always enormous, and, for any town of considerable population has at various times been estimated at thousands of pounds by statisticians. The impediments placed in the way of locomotion are alike disastrous in their effects upon pleasure and upon business ; so that, on the whole, it may fairly be concluded that the expense of a dense fog to a populous community is scarcely less serious than that of an epidemic. It is surely time that the former evil should be striven against as strenuously as the latter.

About twenty years have passed away since Mr. John Aitken, of Falkirk, showed that fog consists of water deposited from the atmosphere upon nuclei furnished by suspended particles. An interesting series of experiments has been conducted at Glasgow, under the direction of Sir John Ure Primrose, the Lord Provost, for the purpose of ascertaining approximately the amount and the character of the particles suspended in the air overhanging that city. In each of the twenty-five wards a water-tight box twelve inches square was placed upon the roof of a building at a height of thirty or forty feet from the ground. The boxes were placed in position on the 1st of March, and were left until the 8th of June, a period of one hundred days, when they were carefully removed and placed in the hands of the corporation chemist for examination. He divided the solid matter deposited in each box into three parts, the mineral or indestructible matter, the oily or greasy matter, and the organic matter. The results obtained naturally differed in the several wards according to their respective densities of population and to their industrial or residential character, as well as, possibly, in a manner influenced by the set of prevailing winds ; but, taking the city as a whole, the average deposit of the one hundred days was found to amount to 109 grains upon each square foot. Of this, 63·5 grains were mineral or incombustible, 3·5 grains were grease or oily matter obtained from the soot, and 42 grains were of smuts and other combustible substances. It was calculated that the annual fall would amount to 22·119 hundredweights on each acre per annum, and that 9·222 hundredweights of this total would consist of combustible material which had escaped combustion, and had been carried into the air by the draught of chimneys. It is fair to assume that particles of this class would be specifically lighter than mineral particles, that they would be more inclined to float, and that they would be more enduring and more available, so to speak, than mineral particles as nuclei on which the water of fog could be deposited.

Sir John Primrose has certainly established that the material of fog must be largely supplied by the results of imperfect combustion ; and he has strenuously applied himself, alike as a manufacturer and as a magistrate, by precept as well as by example, to diminish what is known as the "smoke nuisance" in the great city over which he bears sway. He makes the exceedingly good and practical suggestion that, whenever a manufacturer or employer can prove that his furnaces and other arrangements for the consumption of smoke are efficient and satisfactory, the penalty for smoke production should not fall upon him, but upon the individual stoker who was in charge of the fire at the time when the offence was committed. Sir John himself pays his firemen an extra shilling a week all the year round, under a contract that the men themselves shall pay all fines for smoke ; but this extra payment ought not to be required, and careless workmen ought to be liable to direct punishment for negligence affecting the health and comfort of the community. Sir John dwells hopefully upon the increase of gas fires for all domestic uses ; but it must be remembered that any gas which is imperfectly purified from sulphur furnishes an enormous invisible particulate residue, which, although it does not colour the resulting fog, supplies an abundance of nuclei on which it may be deposited. The fog from which we have lately suffered in England has been by no means exclusively urban ; and it seems highly probable that the abundant dust raised from country roads by the all-pervading motor car may have been to some extent responsible for its formation.

